# *Evernia* Goes to School: Bioaccumulation of Heavy Metals and Photosynthetic Performance in Lichen Transplants Exposed Indoors and Outdoors in Public and Private Environments

**DOI:** 10.3390/plants8050125

**Published:** 2019-05-13

**Authors:** Luca Paoli, Zuzana Fačkovcová, Anna Guttová, Caterina Maccelli, Katarína Kresáňová, Stefano Loppi

**Affiliations:** 1 Department of Biology, University of Pisa, Via L Ghini, 13-56126 Pisa, Italy; luca.paoli@unipi.it; 2 Plant Science and Biodiversity Centre, Slovak Academy of Sciences, Dúbravská Cesta, 9-84523 Bratislava, Slovakia; zuzana.fackovcova@savba.sk (Z.F.); anna.beresova@savba.sk (A.G.); 3 Department of Life Sciences, University of Siena, Via PA Mattioli, 4-53100 Siena, Italy; caterina.maccelli@student.unisi.it; 4 Spojená škola Tilgnerova, 714/14 Karlova Ves, 84105 Bratislava, Slovakia; kaliakatka@gmail.com

**Keywords:** biomonitoring, chlorophyll fluorescence, citizen science, *Evernia prunastri*, exposed to control ratio, indoor pollution

## Abstract

Recently indoor air quality (IAQ) has become a key issue, especially in schools, where children spend most of the day. Only in a few cases IAQ was investigated using lichens as biomonitors. During autumn 2017, lichens (*Evernia prunastri*) were exposed for two months indoors and outdoors in public (schools) and private (dwellings) environments, in both rural and urban areas of Slovakia. The bioaccumulation of selected elements and the physiological status of the samples were considered. The content of heavy metals increased in samples exposed outdoors for 11 out of 12 elements (Al, As, Cd, Cr, Cu, Fe, Pb, S, Sb, V and Zn, but not Ca) in the urban area and for 5 (As, Cd, Cu, Pb and Sb) in the rural area. Indoor concentrations were overall similar, both in rural and urban buildings, independently of the outdoor conditions. An indoor accumulation occurred only for Cd, Cu and Pb. An indoor origin was suggested for Cd, while for Cu and Pb, outdoor penetration (car traffic) is the likely cause of indoor values. Indoor exposed lichens maintained their vitality (as reflected by chlorophyll *a* fluorescence emission). This latter result further supports the use of lichen biomonitoring as a suitable method for assessing IAQ.

## 1. Introduction

The quality of the air that we constantly breathe is more relevant for our health than we think, and recent estimates showed that 92% of the world’s population (urban and rural) lives in places with air pollution levels exceeding WHO guidelines [1]. In any environment, we inevitably inhale the surrounding air, with airborne compounds coming in contact with our cells and impacting our life and health. Usually, we perceive air pollution as an outdoor phenomenon; however for many pollutants, the intake per unit of indoor emission may be several orders of magnitude higher than that of outdoor emissions [2]. In addition, most people spend over 85% of their time within indoor environments, such as schools, dwellings, transports, shops, restaurants, offices and working places in general. Hence, the quality of the air in indoor environments is of paramount importance for our personal well-being and comfort [3].

There can be several toxic compounds in indoor air, such as VOCs, PAHs, NO_2_, CO, CO_2_ and heavy metals, which are mainly originated from human activities, cleaning products, furniture materials, heating and from outdoor infiltration such as traffic pollution, industrial emissions, etc. [4]. Such sources can lead to the accumulation of indoor pollutants, increasing the risk of asthma, rhinitis, allergies or even cancer for those people exposed for long periods [4]. Children are particularly susceptible to indoor air quality (IAQ), since they inhale higher volumes of air than adults in relation to their body weight [5], thus making monitoring of their environments of particular concern [6].

In this sense, specific studies characterized and quantified indoor air pollutants in school environments [7,8,9,10,11,12,13], whereas other studies showed relationships between indoor air quality in schools and health problems (such as rhinitis and asthma) in students and teachers [14,15]. The research has highlighted the need to monitor air quality in school environments, with the aim of controlling the exposure of children to air pollutants and understanding the best measures to be taken to improve air quality in order to obtain safer, more productive and more comfortable conditions for students and school employees.

Biological monitoring (biomonitoring), using sensitive organisms such as lichens, allows an early detection of the biological effects of pollutants that cannot be measured by any non-living item, such as the bioaccumulation of persistent pollutants (e.g., heavy metals), and changes in physiological parameters (indicators of vitality), thus providing information not only on trends in ambient pollution burdens but also on their effects on ecosystems [16,17]. 

Lichens, symbiotic associations between fungi, algae and/or cyanobacteria, are able to take up atmospheric pollutants, especially heavy metals in particulate forms, from the surrounding environment [18,19]. In the case of passive uptake mechanisms, extracellular accumulation can occur irrespective of sample vitality [20,21], while active uptake is linked to lichen metabolism, and hence to the sample vitality [22].

Lichens have been widely used as biomonitors of outdoor air quality; however, only in recent years have some applications been related to IAQ, such as in the case of schools [23,24,25]. Biomonitoring techniques can elegantly integrate instrumental (chemico-physical) sampling methodologies, identify pollution sources and assess outdoor contribution to IAQ. For example, classrooms with potentially worse IAQ can be identified by a higher accumulation of inorganic pollutants in exposed biomonitors [24].

The present research was carried out to investigate IAQ using lichen transplants (*Evernia prunastri* (L.) Ach.) as biomonitors in public (schools) and private (houses) environments. To this purpose, the bioaccumulation of selected trace elements and the physiological status of the transplants were investigated indoors and outdoors in an urban and a rural area of Slovakia. Our working hypothesis is that lichens exposed outdoors in the urban area accumulate higher levels of inorganic contaminants. Therefore, missing an indoor source, IAQ should be influenced by outdoor pollution. Under these conditions, we i) tested the contribution of outdoor pollution by heavy metals to indoor air quality; ii) compared public (schools) with private (dwellings) environments of the same area; and iii) assessed whether the vitality of indoor exposed samples is comparable to those outdoors and hence, the feasibility of using transplants of the lichen *Evernia prunastri* to monitor IAQ.

## 2. Results

Table 1 summarizes raw element concentrations bioaccumulated in the lichen *E. prunastri*, as well as photosynthetic parameters (indicators of vitality) prior to the experiment and after two months of exposure in schools and houses at urban and rural sites. Although higher outdoor concentrations were measured in the urban area, indoor concentrations were on the whole similar both in rural and urban schools and houses, irrespective of the outdoor conditions. 

**Figure 1:**
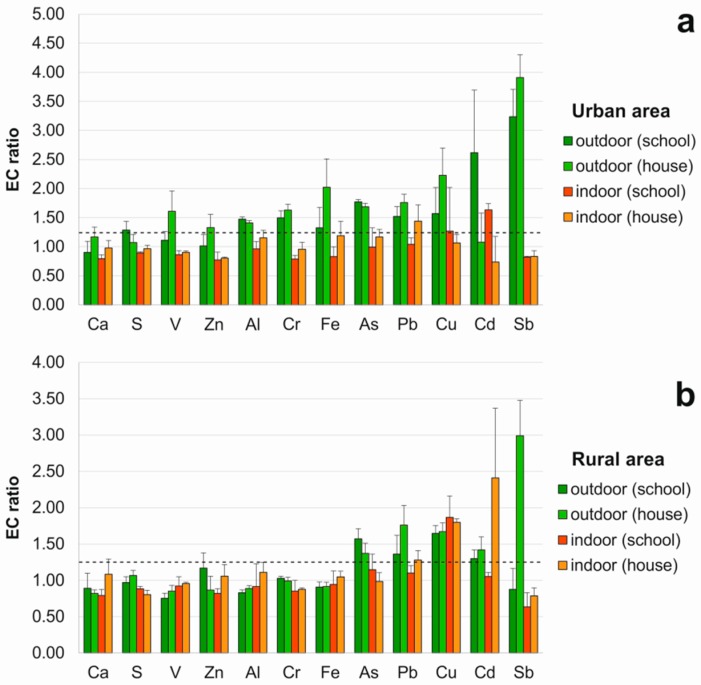
Exposed to Control ratios (average values in vertical bars ± standard deviation) of samples transplanted outdoors and indoors in: (**a**) urban area; (**b**) rural area. The dashed line indicates a significant accumulation (threshold EC > 1.25).

In terms of the ratio between the concentration of each element after and before the exposure (exposed to control—EC—ratios) (Figure 1), outdoor concentrations significantly increased for 11 out of 12 elements (but not Ca) in the urban area (namely Al, As, Cr, Cu, Fe, Pb and Sb, both in the school and the house; Cd and S only in the school; V and Zn only in the house), and for 5 out of 12 elements in the rural area (namely As, Cd, Cu and Pb, both in the school and the house, and Sb only in the house). 

Indoor accumulation (EC > 1.25) occurred only for Cd, Cu and Pb; in the urban area: Cd and Cu in the school, and Pb in the house, and in the rural area: Cu in the school, and Cd, Cu and Pb in the house. Hence, to check for a possible indoor origin of accumulated elements, indoor/outdoor (I/O) ratios were calculated for those elements with EC > 1.25 indoors. The results indicated values >1 only in the rural area for Cd in the house (1.70). For Cu, both in the school (1.13) and the house (1.08), I/O ratios were slightly higher than 1, however indoor and outdoor raw concentrations did not differ significantly (Table 1), therefore an indoor origin of such depositions was not clearly evident. In the case of Pb, an indoor source was readily excluded, since I/O ratios resulted <1 (namely 0.82 in the urban area and 0.73 in the rural area); the same goes for Cd and Cu in the school of the urban area (0.62 and 0.81, respectively). 

Sample vitality was checked by comparing the photosynthetic performance of the samples prior to and after the transplant, and the results (as reflected by chlorophyll *a* fluorescence emission—namely F_V_/F_M_ and PI_ABS_—see Table 1) excluded a significant influence of the exposure conditions. It is noteworthy that, along with the humid season, an increase of the index of the overall photosynthetic performance (PI_ABS_) occurred in all samples exposed outdoors (*p* < 0.05).

## 3. Discussion

The assessment of indoor air quality (IAQ) is of paramount importance in many instances, especially when children are concerned (i.e., in schools). Based on different case-studies, contrasting results may be found in the literature. Yang et al. [26] reported higher levels of particulate matter (PM_10_) indoors than outdoors, with I/O ratios in the range 1.43–2.06 depending on the type of school rooms, with the highest values found in classrooms, and explained this result by the activities of students, such as walking and running during breaks causing the resuspension of particulate matter. Similar results were reported by Almeida et al. [27], who found concentrations of PM_2.5–10_ in classrooms significantly exceeding the ambient levels, and suggested that the physical activity of the pupils led to resuspension of such coarse particles. Previous studies carried out in Poland have shown that indoor concentrations of particulate matter during teaching hours in winter were higher than the outdoor concentrations, suggesting that children could have been subjected to higher particulate matter inside the school, than outside e.g., [8]. On the other hand, Typpayawong et al. [28] showed a significant contribution to indoor particles from penetration of outdoor particles, with I/O ratios in the range 0.69–0.88, and did not find strong evidence of indoor pollution caused by students’ activity. 

We focused our attention on heavy metal depositions and assumed that the content of elements in lichen thalli exposed outdoors and indoors in schools and dwellings reflected the characteristics of their respective environments. Our results indicated a bioaccumulation for Cu in the classrooms of both urban and rural environments and for Cd in the urban area. 

In the case of Cd and Cu in the school of the urban area (I/O ratios < 1, 0.62 and 0.81, respectively) an outdoor contribution to the air pollution burden inside the classrooms is probably linked to vehicular traffic. The same holds true also for Pb at both areas, and as suggested by the I/O ratios (I/O < 1), an outdoor penetration is the most likely cause of indoor levels. An outdoor source could be responsible also for the accumulation of Cu in the rural area, but despite I/O ratios > 1 both in the school (1.13) and the house (1.08), raw concentrations pointed out no difference between indoor and outdoor values. Therefore, an indoor source for Cu depositions was not clearly evident.

Protano et al. [25] transplanted the fruticose lichen *Pseudevernia furfuracea* for two months in five schools of Central Italy, one in a highly urbanized area and four in rural settings, and found higher outdoor concentrations of heavy metals and polycyclic aromatic hydrocarbons in the urban area and an overall bioaccumulation for all elements investigated (As, Cd, Cr, Cu, Hg, Ni and Pb), but I/O ratios > 1 were found only for Cd at the urban school and Hg in the rural area. In our case, we revealed an indoor accumulation limited to Cd, Cu and Pb, in the urban as well as the rural area, which was attributed to vehicular traffic. In fact, such elements (and also Sb, significantly accumulated by outdoor samples) are traffic-related metals, whose content in lichen thalli is regarded as indicator of traffic pollution also in sites with a limited environmental contamination [29,30]. 

Canha et al. [23,24], with a transplant experiment carried out in rural and urban areas of Portugal using the foliose lichen *Flavoparmelia caperata*, compared indoor and outdoor school environments. They found an accumulation for several chemical elements both outdoors and indoors and highlighted Ca as the sole element of indoor origin, likely from the chalk used on blackboards. Furthermore, a partial alteration of cell membranes permeability in exposed thalli was reported, suggesting a possible stress to indoor samples, especially in urban schools closer to the main roads. In our case, Ca was not significantly accumulated, likely due to a limited use of chalks during lectures and thanks to the cleaning of the classrooms, which reduces dust resuspension.

The use of living organisms (plants, mosses and lichens) in the framework of IAQ assessment has only few and recent applications [23,24,25,31,32], with an interesting research focusing on the genotoxic risk in classrooms estimated with the common houseplant *Scindapsus aureus* [31]. In addition to schools, other indoor environments have also been investigated: e.g., Vuković et al. [32] used transplants of the moss *Sphagnum girgensohnii* to study indoor pollution by heavy metals in parking garages of Belgrade (Serbia). The results pointed out an accumulation for outdoor samples. Moss-bags at the garage entrance accumulated higher amounts of heavy metals than those exposed in the interior, allowing to reflect changes on a small-scale, but also suggesting that a lower relative element enrichment within the garages is probably a consequence of the dry indoor environment, which limited both moss physiological activity and a further (ionic) element uptake [32]. Paoli et al. [33] have shown that samples of the lichen *E. prunastri* exposed in smokers’ cars for two months accumulated a relevant amount of heavy metals (Al, As, Cd, Cr, Cu, Ni, Pb and Sb) and nicotine, and that such exposure altered the photosynthetic activity of the thalli [33].

There is an increasing deal of attention also towards IAQ in domestic private environments. Mestl et al. [34] found that the rural population of China was exposed to higher level of indoor air pollution by PM than the urban population, irrespective of the much higher outdoor air particulate level in urban areas. The authors showed that to heat their houses, biomass users were those more exposed, followed by coal users, while gas users were the least exposed. Kim and Fergusson [35] investigated the concentrations and sources of Cd, Cu, Pb and Zn in house dust in Christchurch (New Zealand) and found that the amounts of all the metals were highly correlated with the overall dustiness of the houses, which was found to be mainly caused by carpet wear. It is noteworthy that they found out that the pigmentation of carpets (red, orange or yellow tones), based on Cd-pigments, was consistent with the indoor levels of this element. In our study, although a bioaccumulation of some elements occurred in the houses, only in the case of Cd at the rural site, I/O ratios (>1) suggested potential indoor sources, one of which could be linked to the presence of colored carpets.

Concerning the vitality of the samples, in our case, chlorophyll *a* fluorescence parameters indicated that the lichens were not affected by the indoor exposure and remained on the whole healthy. It is worth noting that an increase of the performance index (PI_ABS_) detected in those samples exposed outdoors was associated with a higher water availability to the samples during autumn (e.g., by precipitation and atmospheric humidity). In addition, the vitality of outdoor transplants (both in urban and rural areas) was comparable to that of the samples prior to the exposure, despite higher concentrations of heavy metals recorded outdoors in the urban area. This finding is in line with the results of Guttová et al. [36] and Lackovičová et al. [37] that recently reported an improved outdoor air quality in the urban area of Bratislava due to a general decline of heavy metal pollution [36] and found out that *E. prunastri* transplants (exposed 6 months) remained overall healthy in large parts of the urban area [37]. 

All previous evidences pointed out the importance of the vitality of the selected biomonitor. In general, indoor samples face an altered light regime, ventilation and a reduced hydration, which should be taken into consideration. To this purpose, we provided an adequate hydration to indoor lichens and the fact that their vitality at the end of the exposure was comparable to those outdoors, suggests that lichen biomonitoring, using adequate methods, can be a suitable tool for the assessment of IAQ.

## 4. Materials and Methods 

### 4.1. Experimental Design and Lichen Material 

The transplant experiment was performed in urban and rural environments of Slovakia. Bratislava, the capital city (ca. 450,000 inhabitants and 200,000 people commuting daily), was selected as the urban area. Intensive traffic, gas heating stations and industrial activities (e.g., engineering, production of chemicals, an oil refinery) are the main local sources of environmental contamination [37]. Madunice, a small village located about 65 km NE of Bratislava (ca. 2,200 inhabitants), was selected as the rural area. Local air pollution is low, related to traffic, heating systems and agriculture. Both in Bratislava and Madunice, the climate is mild, dry or moderately dry, with mild winters [38].

To investigate IAQ in public environments, two schools were selected, one in Bratislava, an elementary and secondary school frequented by about 1,000 students (48°9′20′′ N, 17°3′22′′ E), and another in Madunice, a primary school with kindergarten frequented by about 250 students (48°28′38′′ N, 17°46′33′′ E).

To investigate IAQ in private environments, the houses of two employees (volunteers), one from each school, were chosen. To be included in the study, volunteers had to: 1) live within a short distance (<3 km) from the school; 2) be a non-smoker and eventually share the house only with non-smokers.

To explore the contribution of outdoor pollution to IAQ, at each study site lichen transplants were exposed in parallel indoors (classrooms/living rooms) and outdoors (courtyards/gardens).

The fruticose (shrub-like) lichen *Evernia prunastri* (L.) Ach., a species widely used in biomonitoring studies [39], was collected for the transplant experiment in a remote area far from pollution sources (43°11′58′′ N, 11°21′32′′ E, alt. 310 m a.s.l, Central Italy). The lichen material from this control area has been used also in other biomonitoring studies, since its chemical content reflects background values of unpolluted areas [30,39]. 

**Figure 2:**
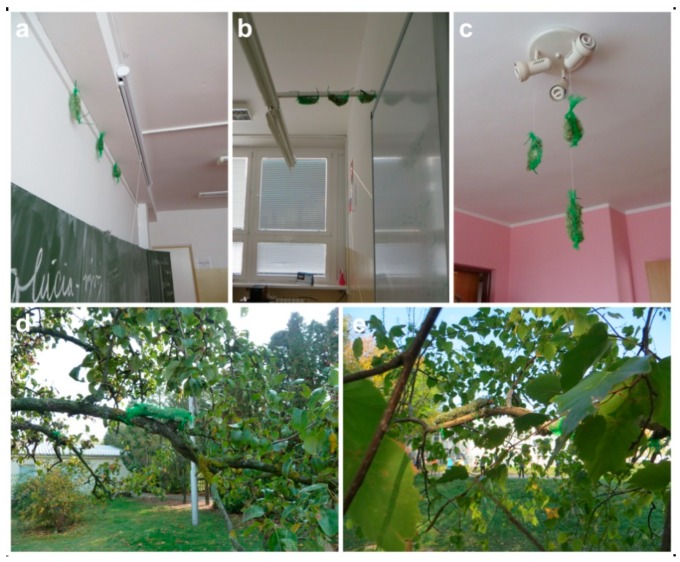
Lichen transplants (*Evernia prunastri*) exposed: (**a**–**c**) indoors; (**d,e**) outdoors.

Prior to the exposure, and after careful manual removal of extraneous materials, lichen samples were washed thrice in deionized water. Samples were exposed using the lichen-bags transplant technique: each transplant was composed of 3–5 thalli (generally 4–5 cm long) gently placed within a plastic net (mesh size 0.8 cm), so that all the material was fully exposed to the surrounding environment. On the whole, 48 lichen-bags (and three controls) were prepared: half of them were placed in the urban area and the other half in the rural area, equally distributed outdoors and indoors. Lichen-bags were bound outdoors to the branches of three independent trees close to the schools and the houses, and indoors (three lichen-bags per room) hanging to the available supports, in both cases at ca. 2 m from ground (Figure 2). The exposure lasted two months (from 19 October to 15 December 2017) since this period proved to be appropriate for the bioaccumulation of elements in lichens transplanted in indoor environments [25]. Samples transplanted indoors were regularly and gently sprayed (not washed) with distilled water thanks to the collaboration of students and teachers, as well as volunteers that provided a regular hydration to the thalli (up to three times per week). For practical reasons, two similar classrooms were selected in each school (for data interpretation data were merged). Schools and dwellings had natural ventilation, generally very limited during the experimental period owing to the cold season. The averages of air temperature and vertical atmospheric precipitation during this period were 5.9 °C, 276 mm in Bratislava, and 5.2 °C, 103 mm in Madunice (data related to schools coordinates; provided by the Slovak Hydrometeorological Institute, Bratislava, Slovakia).

After the exposure period, lichen transplants were retrieved, stored in paper bags at about −18 °C until the analysis in order to keep *E. prunastri* suitable for later physiological and chemical measurements [40]. Control samples were kept in the freezer during the exposure period. Sample vitality (photosynthetic performance), and hence the bioaccumulation capacity, were checked by comparing the samples prior to and after the transplant.

### 4.2. Bioaccumulation of Trace Elements

In the laboratory, lichen thalli were checked under a binocular microscope for extraneous material eventually deposited on the surface. The marginal parts of the laciniae (up to 2.5 cm from lobe tips) were selected for the analysis, being more exposed. This choice is foreseen by the protocols generally applied in the field of biomonitoring with lichens. The laciniae of *E. prunastri* have a wide surface/volume ratio to intercept ambient particulates.

One sample was prepared from each lichen-bag and the lichen material was randomly selected. Samples were minced by plastic tweezers, and the lichen material (about 200 mg) was mineralized with a mixture of 3 mL of 70% HNO_3_, 0.2 mL of 60% HF and 0.5 mL of 30% H_2_O_2_ in a microwave digestion system (Milestone Ethos 900) at 280 °C and 55 bar. The concentrations of selected elements (Al, As, Ca, Cd, Cr, Cu, Fe, Pb, S, Sb, V and Zn) were determined by ICP-MS (Perkin Elmer Sciex, Elan 6100) and expressed on a dry weight basis (µg g^−1^ dw). The analytical quality was checked by the Standard Reference Material IAEA-336 (lichen). A sample of the certified reference material and one procedural blank with the reagents were included in each digestion trial. Recoveries were in the range 95–105%; precision of analysis was expressed by the relative standard deviation of 5 replicates and was below 10% for all elements. Three independent samples were measured for each experimental condition (N = 3).

### 4.3. Assessment of the Vitality of the Samples

In order to evaluate whether the vitality of indoor samples was comparable to the vitality of the samples exposed outdoors, chlorophyll (Chl) *a* fluorescence emission analysis was used. We assessed the photosynthetic performance of the samples by the potential quantum yield of primary photochemistry (F_V_/F_M_), where F_V_ = (F_M_-F_0_) is the variable fluorescence and F_0_ and F_M_ are, respectively, the minimum and maximum Chl *a* fluorescence. In addition, an overall index of the photosynthetic performance, the so called performance index (PI_ABS_), was calculated. In the laboratory, samples were reactivated for 24 hours, sprayed with mineral water and kept humid at 16 °C in dim light (70 µmol m^−2^s^−1^). Prior to the measurements, samples were again sprayed and dark adapted for 10 minutes. The thalli were then lightened for one second with a saturating (3000 µmol m^−2^s^−1^) light pulse and fluorescence emission was recorded for one second. Measurements were carried out with a Plant Efficiency Analyzer (Handy PEA, Hansatech Ltd., Norfolk, UK). Since fluorescence measurements are not destructive, ten measurements were carried out per each lichen-bag and up to three lichen-bags were measured for each experimental condition (N = 30).

### 4.4. Data Interpretation

Non parametric statistics were used. For each experimental condition, the Mann–Whitney U test (*p* < 0.05) was used to check whether a significant accumulation (or variation of photosynthetic parameters) occurred in respect to the control samples. In case of differences, we checked whether samples in the same locality differed according to their indoor/outdoor exposure.

For a standardized representation of the results, the accumulation of chemical elements in the lichen *E. prunastri* was then assessed in terms of the ratio between the concentration of each element after and before the exposure (exposed to control—EC—ratio). The level of loss/accumulation was interpreted according to the scale suggested by Frati et al. [41]: <0.25 severe loss; 0.25–0.75 loss; 0.75–1.25 normal; 1.25–1.75 accumulation; and >1.75 severe accumulation. EC ratios were then used to investigate a possible indoor origin of accumulated elements; to this purpose, indoor/outdoor (I/O) ratios were calculated for those elements with EC > 1.25 indoors.

## 5. Conclusions

The lichen *E. prunastri* has been used to investigate indoor air quality (IAQ) in schools and dwellings in rural and urban areas of Slovakia. We focused our attention on heavy metals depositions and assumed that the content of elements in lichen thalli exposed outdoors/indoors in schools and dwellings reflected the characteristics of their respective environments. Exposed to control (EC) ratios indicated higher heavy metals deposition in the urban area. Nevertheless, indoor concentrations were similar both in rural and urban areas, in schools as well as dwellings, independently of the outdoor conditions, hence suggesting a limited infiltration of air pollution from outdoors. An indoor uptake occurred for a few traffic related elements (Cd, Cu and Pb). The vitality of indoor exposed samples was comparable to those exposed outdoors, suggesting that the lichen *Evernia prunastri* is well suited to be used in the framework of IAQ assessment, when the evaluation of biological accumulation of trace elements is requested. An exposure period of two months was adequate to allow enough time for element bioaccumulation and avoid any physiological impairment of the samples.

## Figures and Tables

**Table 1:** Element concentrations (µg g^−1^, N = 3) and photosynthetic parameters (potential quantum yield of primary photochemistry—F_V_/F_M_ and performance index—PI_ABS_, N = 30) in the lichen *Evernia prunastri* before and after indoor or outdoor exposures (average ± standard deviation). As a first step we checked whether a significant accumulation (or variation of photosynthetic parameters) occurred, and the values in bold are significantly higher than the control samples; in such cases, we checked whether samples in the same locality differ according to their indoor/outdoor exposure (pairs in italics) (Mann–Whitney U test, *p* < 0.05).

Parameters	Pre-Exposure	Urban				Rural			
		School		House		School		House	
		Outdoors	Indoors	Outdoors	Indoors	Outdoors	Indoors	Outdoors	Indoors
Al	445 ± 76	***655 ± 24***	*427 ± 57*	627 ± 12	513 ± 29	369 ± 6	407 ± 140	395 ± 11	494 ± 20
As	0.166 ± 0.011	***0.293 ± 0.026***	*0.164 ± 0.055*	***0.279 ± 0.037***	***0.193 ± 0.019***	***0.260 ± 0.041***	*0.190 ± 0.035*	***0.227 ± 0.030***	*0.163 ± 0.027*
Ca	8083 ± 945	7297 ± 670	6407 ± 533	9431 ± 920	7893 ± 380	7186 ± 520	6394 ± 680	6623 ± 350	8762 ± 642
Cd	0.058 ± 0.005	***0.153 ± 0.014***	***0.095 ± 0.006***	0.063 ± 0.006	0.043 ± 0.007	***0.076 ± 0.004***	*0.061 ± 0.003*	***0.083 ± 0.008***	***0.141 ± 0.022***
Cr	1.13 ± 0.21	***1.69 ± 0.12***	*0.89 ± 0.07*	***1.84 ± 0.05***	*1.08 ± 0.05*	1.16 ± 0.08	0.96 ± 0.17	1.12 ± 0.09	0.99 ± 0.03
Cu	4.0 ± 0.4	***6.2 ± 0.2***	***5.0 ± 0.3***	***8.8 ± 0.7***	*4.2 ± 0.1*	**6.5 ± 0.5**	**7.4 ± 1.2**	**6.6 ± 0.7**	**7.1 ± 0.4**
Fe	342 ± 22	***453 ± 29***	*283 ± 58*	***691 ± 16***	***406 ± 29***	310 ± 5	323 ± 64	313 ± 20	358 ± 16
Pb	1.25 ± 0.07	***1.87 ± 0.04***	*1.30 ± 0.14*	***2.20 ± 0.11***	***1.81 ± 0.07***	***1.74 ± 0.02***	*1.37 ± 0.13*	***2.23 ± 0.07***	***1.59 ± 0.07***
S	816 ± 81	***1049 ± 16***	*727 ± 15*	874 ± 17	788 ± 17	791 ± 3	721 ± 26	870 ± 28	656 ± 26
Sb	0.044 ± 0.004	***0.144 ± 0.007***	*0.036 ± 0.004*	***0.174 ± 0.011***	*0.037 ± 0.005*	0.039 ± 0.004	0.028 ± 0.009	***0.133 ± 0.024***	*0.035 ± 0.003*
V	1.040 ± 0.086	1.154 ± 0.108	0.895 ± 0.073	***1.672 ± 0.043***	*0.936 ± 0.041*	0.782 ± 0.041	0.959 ± 0.133	0.885 ± 0.072	0.993 ± 0.106
Zn	23.3 ± 2.4	23.6 ± 1.9	18 ± 3.1	***30.9 ± 1.1***	*18.7 ± 0.9*	***27.2 ± 1.7***	*19.1 ± 1.5*	20.2 ± 1.7	24.6 ± 1.2
F_V_/F_M_	0.700 ± 0.072	0.765 ± 0.041	0.720 ± 0.051	0.775 ± 0.041	0.722 ± 0.050	0.757 ± 0.035	0.690 ± 0.065	0.766 ± 0.037	0.697 ± 0.053
PI_ABS_	0.165 ± 0.085	***0.294 ± 0.103***	*0.195 ± 0.078*	***0.369 ± 0.066***	*0.197 ± 0.063*	***0.271 ± 0.064***	*0.157 ± 0.055*	***0.275 ± 0.098***	*0.165 ± 0.047*
